# Tobacco-Use Susceptibility and Ever-Use Categories in Relation to Depressive Symptoms Among Undergraduates at a Chinese University: A Cross-Sectional Study

**DOI:** 10.3390/healthcare14172876

**Published:** 2026-09-07

**Authors:** Yu Chen, Xinjie Zhao, Bin Qi, Shiyu Liu, Chengbin Wu

**Affiliations:** 1 School of Art and Communication, Fujian Polytechnic Normal University, Fuqing 350300, China; 2 School of Journalism and Communication, Peking University, Beijing 100871, China; 3 Department of Vocational Education Development, Fujian Polytechnic Normal University, Fuqing 350300, China; 4 School of Public Health, Xi’an Jiaotong University, Xi’an 710061, China; 5 Chongqing Health Education Institute, Chongqing 401120, China

**Keywords:** tobacco susceptibility, e-cigarettes, depressive symptoms, life satisfaction, China

## Abstract

**Highlights:**

**What are the main findings?**

- Depressive symptoms showed a graded cross-sectional pattern across tobacco uptake/susceptibility categories and were already elevated among ‘never users’ who lacked a firm decision not to use tobacco.
- Distress tracked current susceptibility rather than having tried tobacco: ‘ever users’ who remained susceptible scored 4.82 CES-D-10 points above firmly non-susceptible never users, whereas those who were firmly non-susceptible did not differ from them.

**What are the implications of the main findings?**

- Brief susceptibility items used in campus tobacco surveys may help identify students with possible unmet mental health needs before any reported tobacco use.
- Campus tobacco-prevention and mental-health services could pilot reciprocal screening and referral with minimal additional survey burdens, while protecting confidentiality and avoiding stigmatization.

**Abstract:**

**Background/Objectives:** Tobacco involvement and depression frequently co-occur among young people, but Chinese campus studies rarely examine mental health across tobacco susceptibility and use categories. We examined cross-sectional associations of three mutually exclusive categories (firmly non-susceptible ‘never users’, susceptible never users, and ‘ever users’) with depressive symptoms and life satisfaction. **Methods:** A cross-sectional survey of 415 undergraduates (75.4% female) was conducted in southeastern China in December 2025. Primary analyses used trend tests and adjusted regression for depressive symptoms; secondary analyses examined susceptibility components and a descriptive bootstrap decomposition of the life-satisfaction association; sensitivity analyses evaluated alternative specifications, model diagnostics, and response quality. **Results:** Mean CES-D-10 scores were 9.29, 11.55, and 13.06 across the three categories (trend *p* < 0.001), and probable depression prevalence was at 46.5%, 60.7%, and 68.1% *(p* = 0.002). Each one-category increase was associated with a 1.74-point-higher adjusted CES-D-10 score (HC3 95% CI: 0.76 to 2.73) and 65% higher adjusted odds of probable depression (95% CI: 1.17 to 2.33). In a descriptive cross-sectional decomposition, the indirect association with lower life satisfaction through concurrent depressive symptoms was −0.071 (bootstrap 95% CI: −0.124 to −0.027); this does not establish causal mediation. Ever users who remained susceptible scored 4.82 points (HC3 95% CI: 2.63 to 7.00) above firmly non-susceptible never users, whereas the 12 non-susceptible ever users did not differ detectably. **Conclusions:** The findings identify a cross-sectional mental-health gradient across tobacco susceptibility/use categories, without establishing temporal direction. Replication in longitudinal, multi-site samples is required before reciprocal screening and referral are implemented at scale.

## 1. Introduction

Tobacco use remains the leading preventable cause of death worldwide [1], and China—home to more than 300 million smokers—carries a disproportionate share of this burden [2]. The Healthy China 2030 agenda targets a reduction in adult smoking prevalence to below 20% [3], and young adults are pivotal to this goal: the first nationally representative survey of Chinese college students reported a current-smoker rate of 7.8% and an ‘ever’ e-cigarette use rate of 10.1% [4], while e-cigarette awareness on Chinese campuses exceeds 90% [5]. Because most regular tobacco use is established during adolescence and early adulthood, the university years represent a decisive window in which trajectories toward or away from nicotine dependence are consolidated [4,6].

At the same time, Chinese universities face a substantial mental health challenge. Meta-analyses estimate that roughly one quarter of Chinese university students report depressive symptoms [7,8], and surveillance indicates that depressive-symptom detection among Chinese young people has remained high [9]. International evidence supports several non-exclusive explanations for the tobacco–-depression association. Under self-medication or affect-regulation accounts, distressed young people may become more open to nicotine because they expect relief, relaxation, or improved concentration; recent longitudinal studies of college students link depressive symptoms and positive affect-reinforcement expectancies to subsequent vaping and other substance-use trajectories [10,11]. In the opposite direction, nicotine exposure, dependence, and the stress of maintaining use may contribute to later mood symptoms; recent longitudinal and quasi-experimental studies have reported associations of vaping or vaping dependence with subsequent depressive or mood problems [12,13]. Shared vulnerabilities, including emotion-regulation difficulties, peer contexts, and other common determinants, may also generate both tobacco involvement and distress [14]. These mechanisms can coexist, and the present cross-sectional data cannot distinguish among them.

A limitation of much of this literature—particularly with respect to China—is that it dichotomizes young people into users and non-users. The smoking-uptake framework distinguishes firmly committed never smokers, susceptible never smokers, experimenters, and more established forms of use [15,16]. Susceptibility—the absence of a firm decision not to smoke—is a strong predictor of later initiation among never-using youth [17,18], and adapted measures predict e-cigarette initiation [19]. For the present cross-sectional analysis, however, we use this framework only to define three mutually exclusive measurement categories: firmly non-susceptible ‘never users’, susceptible never users, and ‘ever users’. These categories summarize status at one survey occasion; they do not represent observed transitions, and participants are not assumed to progress from one category to the next.

Comparing mental health across these categories, rather than across a binary use/non-use split, addresses whether elevated depressive symptoms are confined to students who report tobacco use or are also evident among never users who lack a firm decision not to use. A previous report from this survey found that a highly susceptible latent class of tobacco-naïve students scored higher on the CES-D-10 than a firmly non-susceptible class [20], while a second report characterized knowledge, attitudes, and practices [21]. In both reports, mental health was an unadjusted descriptor of tobacco-defined groups. The present analysis uses the full sample and adjusted models to examine the cross-sectional association directly.

The primary analysis treats depressive symptoms as the dependent variable and tobacco uptake/susceptibility category as the independent variable. A secondary analysis treats life satisfaction as the dependent variable and uses concurrent depressive symptoms as a statistical intermediate variable in a descriptive decomposition. Life satisfaction represents the cognitive component of subjective well-being [22,23] and has been associated with substance use and other health behaviors [24,25,26,27]. Perceived social support was examined as a covariate with a potential compensatory association and, in an exploratory manner, as a possible modifier of the category–depression association [28]. These analytic roles do not imply temporal or causal ordering.

The primary aim was to test whether depressive symptoms and probable depression display a graded cross-sectional association across the three tobacco uptake/susceptibility categories (H1). The secondary aims were to separate ever use from current susceptibility and to quantify the descriptive indirect association between category and life satisfaction through concurrent depressive symptoms (H2). The independent association of perceived social support was also evaluated, while its interaction with the tobacco category was exploratory (H3). Sensitivity analyses assessed alternative coding, product-specific definitions, a stricter depression threshold, additional covariates, functional form, influential observations, survey duration, and identical-option CES-D-10 response patterns. The response-pattern analysis was post hoc. No hypothesis is interpreted as being temporal or causal.

## 2. Materials and Methods

### 2.1. Study Design, Setting, and Participants

This cross-sectional study is reported in accordance with the STROBE statement for observational studies [29]; the completed checklist is provided as Supplementary Table S3. An online survey was administered in December 2025 among undergraduate students at a public teacher-training university in southeastern China, an institution with an approximately 75% female enrollment. Eligible participants were enrolled undergraduates aged ≥18 years. The questionnaire was distributed through an online survey platform via class groups and university social-media channels, supplemented by snowball sharing. According to the fieldwork cleaning log, 492 questionnaires were returned and 77 were excluded (41 for completion time <3 min, 25 for non-undergraduate status, and 11 for >50% missing tobacco items), yielding 415 valid responses (84.35% of returned questionnaires). The retained analytic dataset contained no missing values for any variable used in the reported models, so all 415 participants were entered in complete-case analyses. The released file contains retained records only; the exclusion counts could not be re-derived from that file. Because recruitment was open and chain-referral based, the number invited is unknown and a conventional participation rate cannot be calculated.

This is a secondary analysis of a survey that generated two previous reports: a latent-class analysis of susceptibility among 368 tobacco-naïve students [20] and a knowledge–attitude–practice analysis with ever use as the dependent variable [21]. Neither used adjusted models with depressive symptoms as the dependent variable, examined life satisfaction, or reported the present statistical decomposition; no regression model, figure, or effect estimate is duplicated.

### 2.2. Measures

**Tobacco uptake/susceptibility category (independent variable).** Ever use was assessed by asking whether participants had ever tried cigarettes or e-cigarettes, even one or two puffs (yes/no for each product). Susceptibility was measured with the validated Pierce algorithm [17], adapted for both products: three parallel items per product (trying in the next 12 months; accepting from a best friend; and use at any time in the future), each rated ‘definitely not’, ‘probably not’, ‘probably yes’, or ‘definitely yes’. A participant was classified as susceptible if any of the six items received a response other than ‘definitely not’. Participants were classified into Level 0, firmly non-susceptible never users (*n* = 312); Level 1, susceptible never users (*n* = 56); or Level 2, ever users of either product (*n* = 47). The numeric levels provide an ordinal coding for a cross-sectional gradient and do not denote temporal progression. Ever use took precedence: respondents who had tried either product were assigned to Level 2 irrespective of current susceptibility, and 12 of the 47 ever users were non-susceptible. More detailed use categories could not be identified because recency and lifetime quantity were not assessed. The variable was modeled as an ordinal linear term in the primary analysis and as indicator variables in a sensitivity analysis.

**Depressive symptoms (dependent variable in the primary analysis; statistical intermediate variable in the secondary decomposition):** The 10-item Center for Epidemiologic Studies Depression Scale (CES-D-10) [30] assessed symptoms over the past week (0–3 per item after recoding; two positively worded items reverse-scored; total range 0–30). The Chinese CES-D-10 has shown its strongest factorial validity in non-clinical college students [31]. A Chinese short form derived from a nationally sampled adult population reported internal consistency of 0.85–0.88, correlations of 0.94–0.96 with the full scale, and proposed 10 as a threshold for depressive tendency [32]. Scores ≥ 10 were therefore used to indicate probable clinically significant depressive symptoms [30,32]; a cut-off of ≥12 was examined in sensitivity analysis because the ≥10 threshold is liberal and was derived outside of a student population. Internal consistency in this sample was good (Cronbach’s α = 0.879). In the secondary cross-sectional decomposition, the CES-D-10 score is a statistical intermediate variable only, not a causal mediator.

**Life satisfaction (dependent variable in the secondary decomposition):** Global life satisfaction was assessed with a single item—‘Overall, how satisfied are you with your studies and life?’—rated from 1 (very dissatisfied) to 5 (very satisfied), consistent with single-item measures used in large surveillance systems [26]. It was interpreted as the cognitive component of subjective well-being [22,23], not as the distinct eudaimonic construct of psychological well-being.

**Perceived social support (psychosocial resource):** A five-item measure compiled for the parent survey [21] assessed perceived adaptability, problem-solving capacity, friend support, family support, and confidant availability (1 = completely untrue to 5 = completely true; total 5–25). This brief measure is not a standardized instrument, and no independent validation is available; it is therefore interpreted as an index of self-reported perceived support rather than as a validated scale, although internal consistency in this sample was high (Cronbach’s α = 0.941), reflecting the homogeneity of these closely related, uniformly positively worded items rather than broad coverage of the construct.

**Covariates:** Sex, academic year (1–4), household residence (urban/rural), any alcohol consumption, meeting the World Health Organization aerobic physical activity recommendation (≥150 min/week moderate or ≥75 min/week vigorous; yes/no), close friends’ cigarette smoking (any vs. none), and parental cigarette smoking (any vs. none) were selected a priori based on prior evidence of associations with tobacco uptake and youth mental health [21,33,34]. In sensitivity analyses, two further variables were additionally adjusted: bar visits in the past 3 months (yes/no) and the count of tobacco-control information channels to which students reported exposure in the past 30 days (range 0–8: billboards/posters, professional health websites, medical institution platforms, social media platforms, short-video platforms, traditional media, school health education, and family/friends).

### 2.3. Statistical Analysis

Analyses were organized as primary, secondary, exploratory, and sensitivity analyses. The primary analysis compared characteristics across the three tobacco uptake/susceptibility categories using chi-squared tests and one-way ANOVA, then tested graded associations using linear contrasts for continuous variables and the Cochran–Armitage test for probable depression. Bonferroni-adjusted pairwise comparisons and eta-squared effect sizes were reported. Hierarchical ordinary least squares (OLS) regression modeled CES-D-10 scores: Model 1 entered covariates, Model 2 added tobacco category, and Model 3 added social support; incremental variance (ΔR^2^), F-change statistics, standardized coefficients (β), and unstandardized coefficients were reported. A logistic model estimated probable depression. The ordinal coding assumes equal differences between adjacent categories, so indicator coding was compared with the linear specification using a partial F test. OLS diagnostics included residual-versus-fitted and scale–location plots, Q-Q plots, the Breusch–Pagan and White tests, Ramsey’s regression-equation specification error test (RESET), externally studentized residuals, leverage, Cook’s distances, and variance inflation factors (VIFs). Because the White and RESET tests identified departures in the final model, inferential statements for OLS coefficients use heteroscedasticity-consistent HC3 standard errors and 95% confidence intervals. Robustness was further evaluated by modeling academic year categorically and social support quadratically and by excluding observations with a Cook’s distance >4/N. The F-change and R^2^ statistics remain conventional OLS fit measures. The retained analytic dataset contained no missing analytic values.

Secondary analyses separated the components of the independent variable—having tried tobacco and current openness to use. Participants were cross-classified by ever use and susceptibility into four groups and entered as indicators with firmly non-susceptible never users as the reference. In addition, the summed six-item susceptibility score (range 6–24) was analyzed as a continuous independent variable within the never-user subsample (*n* = 368), for which the Pierce algorithm was validated. These analyses were secondary rather than confirmatory.

A secondary, descriptive cross-sectional decomposition quantified the total association between tobacco category and life satisfaction, the association between category and concurrent depressive symptoms (component *a*), the association between depressive symptoms and life satisfaction conditional on category (component *b*), the direct component (*c*′), and the product *a* × *b*. All regression equations adjusted for the covariates and social support. Regression-component inference used HC3 standard errors; percentile confidence intervals for a × b used 5000 nonparametric bootstrap resamples with a fixed reproducibility seed [35,36]. The ratio (*a* × *b*)/*c* was calculated descriptively but not emphasized because it is unstable when the total association is imprecise. Because all three variables were measured concurrently, the temporal-ordering and no-unmeasured-confounding assumptions required for causal mediation cannot be verified [35]. Accordingly, *a* × *b* is termed an indirect association, depressive symptoms a statistical intermediate variable, and the decomposition descriptive; terms such as ‘full mediation’ or ‘causal pathway’ are avoided [37].

Sensitivity analyses (a) used indicator coding for tobacco category, (b) constructed cigarette- and e-cigarette-specific variables, (c) used Spearman correlation for the ordinal life-satisfaction variable, (d) additionally adjusted for bar visits and tobacco-control information exposure, (e) applied the more conservative CES-D-10 threshold of ≥12, (f) evaluated flexible functional form and influential observations, (g) excluded the fastest 5% and 10% of completions, and (h) re-estimated models after excluding respondents who selected the same raw response option for all ten CES-D-10 items. This zero-within-person-variation pattern is compatible with straightlining or low response differentiation, particularly because the scale mixes positive- and negative-valence items, but it does not by itself prove inattention, logical inconsistency, or invalidity; positive and negative affect can co-occur, and respondents may interpret item wordings imperfectly. The identical-option exclusion was therefore post hoc and was not used to define the primary sample. Analyses used Python 3.12.13 (Python Software Foundation, Beaverton, OR, USA), with NumPy 2.3.5, SciPy 1.17.0, and pandas 2.2.3; reproducible matrix calculations and bootstrap code are supplied with the revision. Two-tailed *p* < 0.05 denoted statistical significance.

### 2.4. Questionnaire Pilot Testing

Before the main survey, the questionnaire was administered in a pilot wave for instrument evaluation (21 November–1 December 2025; *n* = 206 undergraduates at the same institution). In that wave, the CES-D-10 showed good internal consistency (Cronbach’s α = 0.851), sampling adequacy (Kaiser–Meyer–Olkin [KMO] = 0.84) and factorability (Bartlett’s test of sphericity *p* < 0.001), and the five-item social support measure performed comparably (α = 0.886; KMO = 0.81; Bartlett’s *p* < 0.001); full results are given in Supplementary Table S1.

The pilot and main waves were recruited from the same institution through the same class-level messaging channels ten days apart, and participation overlapped: at least 43 respondents in each wave could be matched to the other by a distinctive survey-platform display name, and a further subset shared submission IP addresses. The two waves are therefore not independent samples, and both indicators are released with the data so that the extent of overlap can be inspected. Because of this overlap, the pilot wave is used exclusively to document the measurement properties of the instruments; it is not considered an independent replication sample, and no substantive association reported in Section 3 draws on it. The pilot data were not pooled with the main survey, and all analyses in this paper use the 415 main-survey respondents only.

### 2.5. Ethical Considerations

Participation was voluntary and anonymous, and electronic informed consent was obtained before completion. The study was conducted in accordance with the Declaration of Helsinki. The Research Ethics Committee of Fujian Polytechnic Normal University approved the protocol (Approval No. 2025-07; 19 November 2025).

## 3. Results

### 3.1. Sample Characteristics Across Tobacco Uptake/Susceptibility Categories

Of the 415 participants (75.4% female), 312 (75.2%) were firmly non-susceptible never users, 56 (13.5%) were susceptible never users, and 47 (11.3%) were ever users of cigarettes and/or e-cigarettes (Table 1). Overall, the mean CES-D-10 score was 10.03 ± 6.14, and 211 students (50.8%) met the ≥10 threshold for probable depression; mean social support was 18.84 ± 4.40, and 78.1% reported being satisfied or very satisfied with their studies and life. Male sex, urban residence, alcohol consumption, bar visits, and having close friends who smoke differed across the categories (all *p* < 0.01), whereas academic year, physical activity, and parental smoking did not differ (Table 1). These are cross-sectional comparisons and do not represent transitions between levels.

### 3.2. Primary Analysis: Graded Cross-Sectional Association with Depressive Symptoms

**Figure 1:**
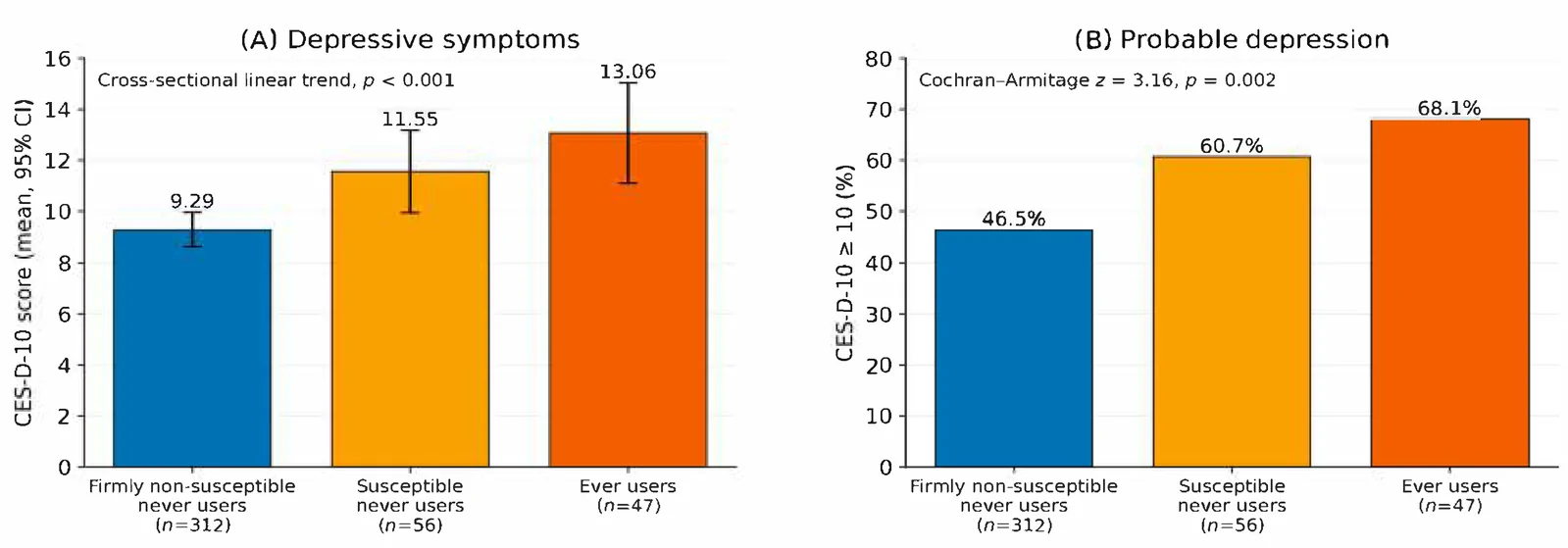
Depressive symptoms across tobacco uptake/susceptibility categories among undergraduate students (*N* = 415). (**A**) Mean CES-D-10 scores with 95% confidence intervals. (**B**) Prevalence of probable depression (CES-D-10 ≥ 10). Levels are cross-sectional categories and do not denote temporal progression.

Depressive symptoms showed a graded cross-sectional pattern across the ordered categories: 9.29 ± 5.89 in firmly non-susceptible never users, 11.55 ± 6.04 in susceptible never users, and 13.06 ± 6.72 in ever users [*F*(2, 412) = 10.12, *p* < 0.001, η^2^ = 0.047; linear trend *t* = 4.01, *p* < 0.001] (Table 2, Figure 1A). Bonferroni-adjusted comparisons showed higher scores in susceptible never users (*p* = 0.027) and ever users (*p* < 0.001) than in firmly non-susceptible never users; the two higher categories did not differ. Probable depression displayed the same pattern (46.5%, 60.7%, and 68.1%; Cochran–Armitage *z* = 3.16, *p* = 0.002) (Figure 1B). Life satisfaction differed among categories [4.17 ± 0.80, 3.82 ± 0.88, and 3.91 ± 1.19; *F*(2, 412) = 4.99, *p* = 0.007, η^2^ = 0.024], although its linear trend was not significant (*p* = 0.060). Social support did not differ significantly and was retained as a covariate and psychosocial resource. Life satisfaction correlated inversely with depressive symptoms (Spearman ρ = −0.37, *p* < 0.001).

### 3.3. Multivariable Models of Depressive Symptoms

In hierarchical regression (Table 3), covariates explained 6.2% of CES-D-10 variance (Model 1). Adding the tobacco category improved fit [ΔR^2^ = 0.036, *F*-change(1, 406) = 16.37, *p* < 0.001], and adding social support produced a further improvement [ΔR^2^ = 0.063, *F*-change(1, 405) = 30.60, *p* < 0.001]. In Model 3 (R^2^ = 0.162), each one-category increase was associated with a 1.74-point-higher CES-D-10 score (HC3 SE = 0.50; 95% CI: 0.76 to 2.73; β = 0.19, *p* < 0.001), while the average linear social-support coefficient was −0.36 (HC3 SE = 0.08; 95% CI: −0.52 to −0.20; β = −0.26, *p* < 0.001). Higher academic year was also associated with higher scores; the close-friend smoking interval narrowly included zero under HC3 inference (*p* = 0.053). Indicator coding did not fit better than linear coding [*F*(1, 404) = 0.08, *p* = 0.775]. Diagnostics identified heteroscedasticity (White F = 1.70, *p* = 0.004) and functional-form departure (RESET F = 11.05, *p* = 0.001), although the Breusch–Pagan test was borderline (*p* = 0.058) and VIFs were ≤1.23. A flexible model with categorical academic year and a quadratic social-support term removed the RESET signal (*p* = 0.840) and yielded a similar category coefficient (b = 1.70; HC3 95% CI: 0.74 to 2.67). Only one externally studentized residual exceeded |3|; the maximum Cook’s distance was 0.037, and excluding the 26 observations above 4/N yielded b = 1.90 (95% CI: 1.11 to 2.69). Full diagnostics are reported in Table S4 and Figure S1. In logistic regression, each one-category increase corresponded to a 65% increase in the adjusted odds of probable depression (OR = 1.65; 95% CI: 1.17 to 2.33; *p* = 0.004); this is an increase in odds, not a 65% increase in probability. The category × social-support interaction was not significant under HC3 inference (*b* = 0.10, SE = 0.13, *p* = 0.431).

### 3.4. Secondary Analysis: Separating Ever Use from Current Susceptibility

Cross-classifying ever use and susceptibility produced four groups whose depressive symptoms differed [*F*(3, 411) = 8.37, *p* < 0.001] (Table 4). Relative to firmly non-susceptible never users, adjusted CES-D-10 scores were 4.82 points higher among ever users who remained susceptible (HC3 95% CI: 2.63 to 7.00; *p* < 0.001) and 1.58 points higher among susceptible never users (HC3 95% CI: −0.30 to 3.46; *p* = 0.100). Ever users who were firmly non-susceptible did not differ detectably from firmly non-susceptible never users (*b* = 0.32; HC3 95% CI: −4.12 to 4.75; *p* = 0.889); their unadjusted mean (9.83), probable-depression prevalence (41.7%), and mean life satisfaction (4.17) were close to those of firmly non-susceptible never users (9.29, 46.5%, and 4.17). The group of ever users who were firmly non-susceptible was small (*n* = 12), so the result is absence of evidence for elevated distress, not evidence of equivalence.

Consistent with this pattern, the summed susceptibility score was associated with depressive symptoms within the never-user subsample (*n* = 368; *b* = 0.38 per point, HC3 SE = 0.15; 95% CI: 0.09 to 0.67; *p* = 0.011), whereas the binary susceptibility indicator was not (*b* = 1.45, HC3 SE = 0.97; 95% CI: −0.46 to 3.35; *p* = 0.136), for the same subsample and model.

### 3.5. Secondary Analysis: Cross-Sectional Statistical Decomposition for Life Satisfaction

**Figure 2:**
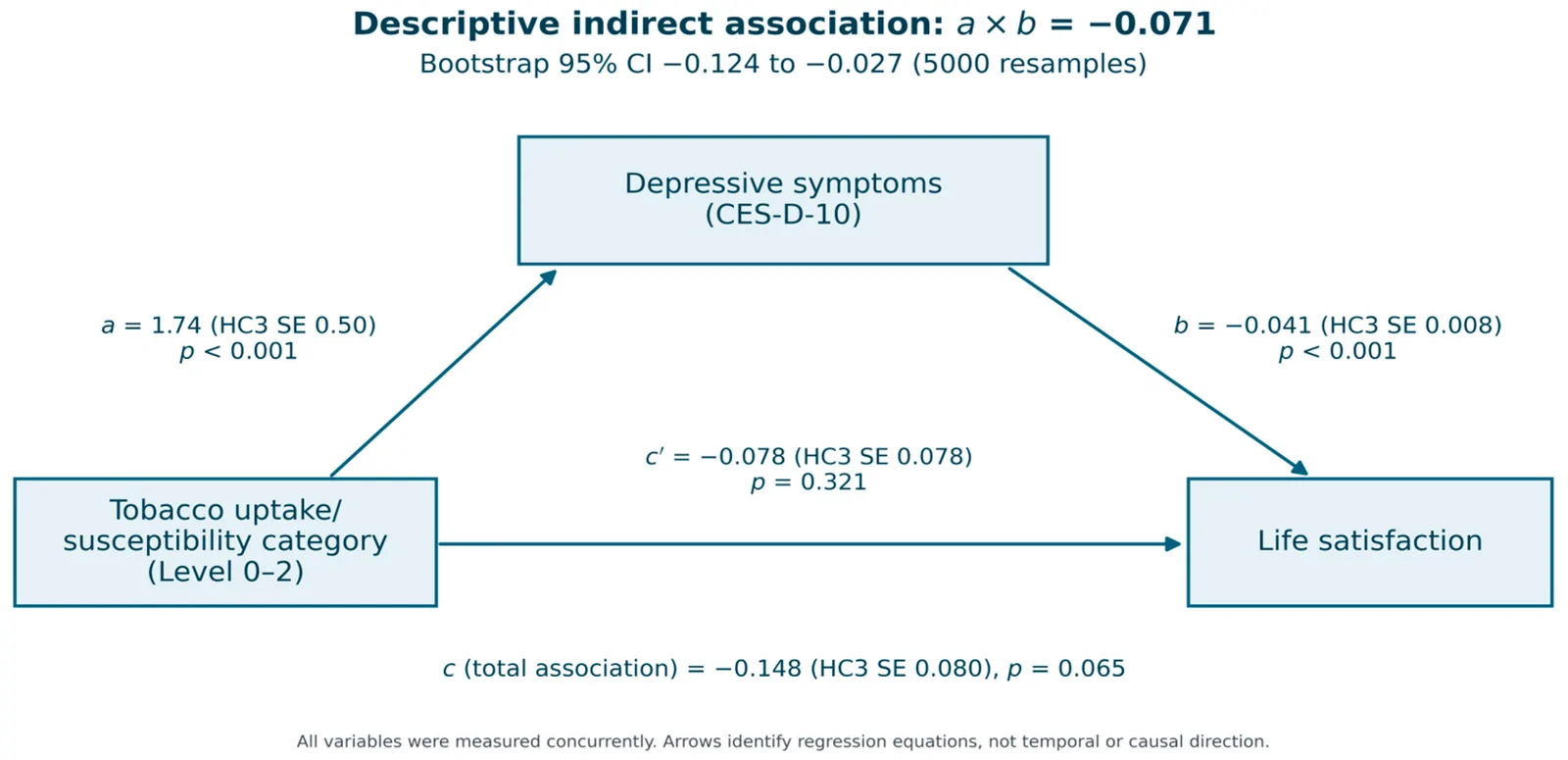
Descriptive cross-sectional statistical decomposition of the association between tobacco uptake/susceptibility category and life satisfaction, adjusted for covariates and social support (*N* = 415). Unstandardized coefficients and HC3 standard errors are shown; the indirect association uses 5000 bootstrap resamples. All variables were measured concurrently; arrows identify regression equations and do not establish temporal or causal direction.

Adjusting for covariates and social support, the estimated total association between a one-category increase and life satisfaction was −0.148 (HC3 SE = 0.080; 95% CI: −0.306 to 0.009; *p* = 0.065) (Table 5, Figure 2). Tobacco category was associated with concurrent depressive symptoms (component *a* = 1.74, HC3 SE = 0.50; 95% CI: 0.76 to 2.73; *p* < 0.001), and depressive symptoms were inversely associated with life satisfaction conditional on category (component *b* = −0.041, HC3 SE = 0.008; 95% CI: −0.056 to −0.025; *p* < 0.001). Their product was −0.071 (bootstrap 95% CI: −0.124 to −0.027), while the direct component was imprecise (*c*′ = −0.078, HC3 SE = 0.078; 95% CI: −0.231 to 0.076; *p* = 0.321). The descriptive ratio (a × b)/c was 47.7% but this is not emphasized, because the HC3 interval for c included zero. This decomposition quantifies concurrent statistical associations only. It cannot show that tobacco involvement preceded depressive symptoms or that depressive symptoms causally reduced life satisfaction; reverse direction and shared causes remain compatible with the data.

### 3.6. Sensitivity Analyses

A cigarette-specific three-category variable (*n* = 318/51/46) reproduced the primary result (*b* = 1.72, HC3 SE = 0.51; 95% CI: 0.72 to 2.73; *p* = 0.001). A corresponding e-cigarette-specific variable was not interpretable, because only one respondent had used e-cigarettes without trying cigarettes; classifying 23 cigarette experimenters as e-cigarette never users yielded non-monotonic category means (9.50, 12.71, and 10.71). Indicator coding produced the same conclusion as ordinal coding. Additional adjustment for bar visits and tobacco-control information exposure changed the coefficient little (*b* = 1.56, HC3 SE = 0.52; 95% CI: 0.54 to 2.58; *p* = 0.003). At the CES-D-10 ≥12 threshold, probable-depression prevalence was 37.6% overall and 32.4%, 50.0%, and 57.4% across Levels 0–2; the adjusted odds ratio was 1.79 per one-category increase (95% CI: 1.28–2.51, *p* < 0.001). Excluding the fastest 5% or 10% of completions also left the category coefficient positive (b = 1.71 and 2.04, respectively; Table S4).

Seventy-two respondents (17.3%) selected the same raw response option for all ten CES-D-10 items, producing zero within-person response variation. This pattern may indicate straightlining, low differentiation, misunderstanding, or acquiescent responding, but it is not definitive evidence of inattention or invalidity. Flagged respondents completed the survey faster (median 257 vs. 357 s; Mann–Whitney *p* < 0.001), and the pattern varied by tobacco category (29.8% of ever users vs. 15.1% of firmly non-susceptible never users; *χ^2^ p* = 0.041). We therefore retained the full sample as primary—post hoc exclusion could introduce differential selection bias—but repeated the models for the remaining 343 participants (Table S2). The category coefficient was essentially unchanged (1.74 vs. 1.73; HC3 95% CIs: 0.76 to 2.73 and 0.69 to 2.78), the adjusted odds ratios strengthened, and the indirect association was stable. The social-support coefficient changed materially, and the estimated direct/indirect allocation shifted; under HC3 inference, however, the direct component remained imprecise in both samples. Thus, the central category–depression association was robust, whereas estimates involving social support and the decomposition require caution.

Two secondary observations emerged. Tobacco-control information exposure differed across the three categories [mean channels: 6.54, 4.88, and 6.17 for Levels 0–2; *F*(2, 412) = 8.77, *p* < 0.001], with susceptible never users reporting the least exposure. Greater information-channel exposure was independently associated with lower depressive symptoms (*b* = −0.23 per channel, SE = 0.10; 95% CI: −0.43 to −0.02; *p* = 0.031).

## 4. Discussion

This cross-sectional study yielded four principal findings. First, depressive symptoms and probable depression displayed a graded association across the three tobacco uptake/susceptibility categories. Second, elevated symptoms were already evident among susceptible never users. Third, secondary analyses suggested that distress was more closely associated with current susceptibility than with ever trying tobacco. Fourth, social support was independently associated with fewer depressive symptoms but did not modify the category–depression association. A descriptive decomposition additionally showed an indirect association between tobacco category and life satisfaction through concurrent depressive symptoms; it is secondary and non-causal.

### 4.1. A Cross-Sectional Mental-Health Gradient Among Never and Ever Users

The most notable finding is the elevation of depressive symptoms among susceptible never users—students who reported no tobacco use but lacked a firm intention to remain tobacco-free. This extends the literature based mainly on user/non-user contrasts [38,39,40] by identifying a mental-health difference within never users. It does not show that susceptibility precedes distress or vice versa. Self-medication and positive reinforcement expectancies could make tobacco more attractive to distressed students [10,11,41]; nicotine exposure or dependence could contribute to later mood problems [12,13]; and shared emotional, social, or contextual vulnerabilities could influence both [14]. The cross-sectional gradient is compatible with all three explanations. Accordingly, susceptibility may be a useful screening signal, but it should not be interpreted as a diagnosis or as evidence that tobacco openness caused distress.

Compared with the earlier unadjusted latent-class analysis of tobacco-naïve students [20], the present adjusted analysis includes ever users and shows that the association persists after accounting for social support, lifestyle factors, and peer and parental smoking. Each category increase corresponded to a 65% increase in the adjusted odds of probable depression. Because the sample is single-site and non-probability-based, the magnitude and screening performance require replication in more diverse student populations.

### 4.2. Openness to Tobacco, Rather than Having Tried It, Tracks Distress

Separating ever use from current susceptibility sharpened the cross-sectional pattern. The 12 non-susceptible ever users resembled firmly non-susceptible never users, whereas susceptible ever users had the highest CES-D-10 scores. Within never users, the continuous susceptibility score was associated with depressive symptoms, while the binary indicator was not. This suggests that degree of openness may carry more information about concurrent distress than a simple yes/no classification. The small non-susceptible ever-user group and wide confidence interval preclude a firm conclusion, and longitudinal research is needed to determine whether changes in susceptibility track changes in mental health.

Practically, a brief susceptibility assessment could complement—not replace—validated mental-health screening. Students should not be singled out solely because they have tried tobacco or expressed uncertainty about future use; any follow-up should be voluntary, confidential, and based on a broader assessment of need.

### 4.3. Concurrent Depressive Symptoms and Life Satisfaction

Tobacco category, depressive symptoms, and life satisfaction were measured concurrently. The secondary decomposition yielded a nonzero bootstrap product through depressive symptoms, but the HC3 interval for the total association included zero and the direct/indirect allocation changed after excluding identical-option CES-D-10 patterns (Table S2). The defensible interpretation is an inverse concurrent association between depressive symptoms and life satisfaction and a reproducible statistical product—not a causal mediation process or a stable mediated proportion. This is consistent with studies linking tobacco involvement and substance use to lower life satisfaction [24,25,26,27], but longitudinal repeated-measures data are needed to establish temporal order and distinguish mediation from shared confounding.

### 4.4. Social Support: Compensatory, Not Buffering

Social support had the largest standardized linear association in the final CES-D-10 model (β = −0.26) but did not modify the tobacco-category association. This resembles a compensatory rather than buffering pattern [28]. The RESET result and flexible model indicated that the support association was nonlinear, and its magnitude changed after excluding identical-option response patterns. Because the index was not independently validated, the linear coefficient should be interpreted as an average association rather than a constant effect across the scale. The result supports evaluating broad, campus-wide support strategies rather than targeting support only by tobacco category.

### 4.5. Implications for Integrated Campus Tobacco Control and Mental Health Services

The findings suggest a testable implementation pathway rather than an immediate policy mandate. Universities could pilot two-way screening in selected settings: add the six susceptibility items to routine mental-health intake and a brief validated depression screen to tobacco-prevention contacts. Positive screens should trigger a private, opt-in assessment by trained staff, not automatic labeling or disciplinary action. Programs should specify referral thresholds, same-day pathways for acute risk, data-minimization and access controls, and staff training in non-stigmatizing communication. Evaluation should measure reach, referral uptake, student acceptability, false-positive burden, and changes in symptoms and tobacco initiation before wider adoption.

A complementary pilot could integrate affect-regulation skills and tobacco-risk content in campus social-media and short-video campaigns, because susceptible never users reported the least exposure to tobacco-control information. Given the cross-sectional design and possible selective attention, this finding should guide message testing rather than be interpreted as evidence that greater information exposure reduces depression. Multi-site cluster or stepped-wedge evaluations could test both effectiveness and unintended effects.

### 4.6. Dual Use Rather than E-Cigarette-Specific Uptake

Cigarette and e-cigarette experience overlapped strongly: among 47 ever users, 23 had tried cigarettes only, 23 both products, and one e-cigarettes alone. This supports the combined definition used here but prevents estimation of an e-cigarette-specific association. Future multi-site studies should recruit larger numbers of exclusive e-cigarette users and measure recency, frequency, and nicotine dependence.

### 4.7. Strengths and Limitations

The strengths include use of a validated susceptibility algorithm to distinguish two never-user categories; a reliable CES-D-10; explicit separation of primary, secondary, exploratory, and sensitivity analyses; assessment of the ordinal specification; decomposition of ever use and susceptibility; updated international context; and transparent differentiation from prior reports based on the survey.

The limitations are substantial. The cross-sectional design precludes temporal or causal inference: distress may increase susceptibility, tobacco exposure may contribute to distress, and shared causes may influence both [10,11,12,13,14,33,35,42]. Convenience and snowball recruitment at one teacher-training university, together with 75.4% female participation, limit generalizability and may bias prevalence. The 50.8% prevalence at CES-D-10 ≥10 exceeds meta-analytic estimates [7,8]; self-selection, sample composition, secular trends, and the liberal screening threshold may contribute limitations. The ≥12 sensitivity analysis produced a lower prevalence (37.6%) but a similar gradient. Life satisfaction was a single item, social support was measured with a non-standardized index, and all variables were self-reported. Seventeen percent of respondents showed an identical-option CES-D-10 pattern. That flag is compatible with low-effort responding but is not diagnostic of invalidity; exclusion did not alter the primary category coefficient, but changed the social-support estimate and decomposition, indicating response-process uncertainty. OLS diagnostics detected heteroscedasticity and nonlinear functional form; HC3 inference and a flexible support specification preserved the category estimate, but the average linear support coefficient and conventional life-satisfaction intervals were sensitive. The four-group analysis included only 12 non-susceptible ever users, ever use combined experimental and continuing use, and small higher-category samples limited interaction power. Unmeasured factors such as academic stress, socioeconomic conditions, prior mental-health history, and personality may confound the associations. These limitations constrain both effect interpretation and any proposed screening implementation.

## 5. Conclusions

Among undergraduates at one Chinese university, depressive symptoms and probable depression displayed a graded cross-sectional association across firmly non-susceptible never users, susceptible never users, and ever users. A descriptive secondary decomposition identified an indirect association with lower life satisfaction through concurrent depressive symptoms, but it did not establish mediation or direction. The elevation among susceptible never users makes susceptibility a candidate signal for integrated campus support, not a causal marker or diagnosis. Universities considering reciprocal tobacco and mental-health screening should first use confidential, opt-in pilots with clear referral pathways and evaluate acceptability, equity, false-positive burden, and student outcomes. Longitudinal, multi-site studies with balanced samples and repeated measures are required before population-wide implementation.

## Figures and Tables

**Table 1:** Characteristics of undergraduate students by tobacco uptake/susceptibility category, southeastern China, December 2025 (*N* = 415).

Characteristic	Level 0: Firmly Non-Susceptible Never Users (*n* = 312)	Level 1: Susceptible Never Users (*n* = 56)	Level 2: Ever Users (*n* = 47)	χ^2^ (df)	*p*
Male sex, *n* (%)	58 (18.6)	18 (32.1)	26 (55.3)	31.72 (2)	<0.001
Urban residence, *n* (%)	161 (51.6)	40 (71.4)	31 (66.0)	9.74 (2)	0.008
Alcohol consumption, *n* (%)	99 (31.7)	32 (57.1)	37 (78.7)	44.90 (2)	<0.001
Bar visit (past 3 months), *n* (%)	23 (7.4)	7 (12.5)	14 (29.8)	21.90 (2)	<0.001
Close friends smoke, *n* (%)	159 (51.0)	41 (73.2)	34 (72.3)	15.05 (2)	<0.001
Parent smokes, *n* (%)	153 (49.0)	28 (50.0)	26 (55.3)	0.64 (2)	0.724
Meets physical activity guideline, *n* (%)	155 (49.7)	28 (50.0)	26 (55.3)	0.52 (2)	0.770
Academic year, χ^2^ across years	—	—	—	1.28 (6)	0.973

Note: Level 0 = firmly non-susceptible never users, answering ‘definitely not’ to all six Pierce items; Level 1 = never users susceptible to cigarettes and/or e-cigarettes; Level 2 = ever users (experimental users) of cigarettes and/or e-cigarettes, even one or two puffs. Levels 0 and 1 jointly comprise the 368 tobacco-naïve students also described in [22]. Chi-squared tests of independence.

**Table 2:** Depressive symptoms, probable depression and life satisfaction by tobacco uptake/susceptibility category (*N* = 415).

Variable	Level 0 (*n* = 312)	Level 1 (*n* = 56)	Level 2 (*n* = 47)	*F*/*z* (Test)	*p*
CES-D-10 score, mean ± SD	9.29 ± 5.89	11.55 ± 6.04	13.06 ± 6.72	*F*(2, 412) = 10.12; η^2^ = 0.047	<0.001
Linear trend (−1, 0, +1)	—	—	—	*t* = 4.01	<0.001
Probable depression (CES-D-10 ≥ 10), *n* (%)	145 (46.5)	34 (60.7)	32 (68.1)	Cochran–Armitage *z* = 3.16	0.002
Life satisfaction (1–5), mean ± SD	4.17 ± 0.80	3.82 ± 0.88	3.91 ± 1.19	*F*(2, 412) = 4.99; η^2^ = 0.024	0.007

Note: CES-D-10 = 10-item Center for Epidemiologic Studies Depression Scale (range 0–30). Bonferroni-corrected pairwise CES-D-10 comparisons: Level 0 vs. 1, *p* = 0.027; Level 0 vs. 2, *p* < 0.001; Level 1 vs. 2, *p* = 0.698. Levels 0 and 1 together comprise the 368 tobacco-naïve students also described in [20]; these two rows are therefore not independent of the descriptive figures reported there.

**Table 3:** Hierarchical linear regression predicting depressive symptoms (CES-D-10).

Predictor	Model 1 *b* (HC3 SE)	Model 2 *b* (HC3 SE)	Model 3 *b* (HC3 SE)	Model 3 95% CI	Model 3 β
Male sex	−0.12 (0.78)	−0.79 (0.78)	−0.84 (0.79)	−2.38 to 0.71	−0.06
Academic year	1.08 (0.29) ***	1.07 (0.28) ***	0.96 (0.28) ***	0.41 to 1.51	0.16
Urban residence	0.63 (0.60)	0.40 (0.58)	0.32 (0.57)	−0.80 to 1.44	0.03
Alcohol consumption	0.67 (0.63)	−0.02 (0.63)	0.30 (0.63)	−0.93 to 1.54	0.02
Meets physical activity guideline	−0.97 (0.65)	−0.83 (0.64)	−0.70 (0.62)	−1.92 to 0.52	−0.06
Close friends smoke	1.39 (0.67) *	1.25 (0.66)	1.27 (0.65)	−0.02 to 2.55	0.10
Parent smokes	0.42 (0.63)	0.42 (0.62)	0.68 (0.59)	−0.47 to 1.84	0.06
Tobacco category (per one-category increase)	—	1.90 (0.50) ***	1.74 (0.50) ***	0.76 to 2.73	0.19
Social support	—	—	−0.36 (0.08) ***	−0.52 to −0.20	−0.26
R^2^	0.062	0.099	0.162	—	—
ΔR^2^ (*F*-change; *p*)	—	0.036 (16.37; <0.001)	0.063 (30.60; <0.001)	—	—

Note: Hierarchical ordinary least squares regression of CES-D-10 scores (*N* = 415). *b* = unstandardized coefficient; HC3 SE = heteroscedasticity-consistent standard error; β = standardized coefficient based on the original variables. R^2^ and F-change are conventional OLS fit statistics. All variance inflation factors ≤1.23. * *p* < 0.05; *** *p* < 0.001.

**Table 4:** Depressive symptoms by cross-classification of ever tobacco use and susceptibility (*N* = 415).

Group	*n*	CES-D-10	≥10, *n* (%)	Life Satisf.	Adjusted *b* (95% CI)	*p*
Firmly non-susceptible never users	312	9.29 ± 5.89	145 (46.5)	4.17	Reference	—
Susceptible never users	56	11.55 ± 6.04	34 (60.7)	3.82	1.58 (−0.30 to 3.46)	0.100
Ever users, non-susceptible	12	9.83 ± 6.39	5 (41.7)	4.17	0.32 (−4.12 to 4.75)	0.889
Ever users, susceptible	35	14.17 ± 6.55	27 (77.1)	3.83	4.82 (2.63 to 7.00)	<0.001

Note: CES-D-10 and life satisfaction are unadjusted means (±SD where shown); ≥10 = probable depression. The first two rows describe the tobacco-naïve subsample also characterized in [20]; the adjusted coefficients and the two ever-user rows are specific to the present analysis. Adjusted coefficients and HC3 confidence intervals are from a single OLS model of CES-D-10 scores including all three indicator variables together with sex, academic year, residence, alcohol consumption, physical activity, close friends’ smoking, parental smoking and social support (model R^2^ = 0.173). Susceptibility is defined by the Pierce algorithm, as applied to the three cigarette and three e-cigarette items; firmly non-susceptible never users answered ‘definitely not’ to all six.

**Table 5:** Descriptive cross-sectional decomposition of the association between tobacco category and life satisfaction (*N* = 415).

Regression Component	Estimate (HC3 SE)	95% CI	*p*
*a*: Tobacco category–depressive symptoms association	1.74 (0.50)	0.76 to 2.73	<0.001
*b*: Depressive symptoms–life satisfaction association	−0.041 (0.008)	−0.056 to −0.025	<0.001
*c*: Total tobacco category–life satisfaction association	−0.148 (0.080)	−0.306 to 0.009	0.065
*c*′: Direct component	−0.078 (0.078)	−0.231 to 0.076	0.321
*a* × *b*: Indirect association	−0.071	−0.124 to −0.027 (bootstrap)	—
Descriptive ratio (a × b)/c; not emphasized	47.7%	—	—

Note: All regression equations adjusted for sex, academic year, residence, alcohol consumption, physical activity, close friends’ smoking, parental smoking, and social support. Components *a*, *b*, *c* and *c*′ use HC3 standard errors and confidence intervals; the indirect association uses a percentile interval from 5000 nonparametric bootstrap resamples (seed 20260806). The ratio (a × b)/c is descriptive and unstable when c is imprecise. Arrows in Figure 2 identify regression equations and do not establish temporal or causal effects.

## Data Availability

The data presented in this study are available from the corresponding author (C.W., yu0300@sina.com) upon request. The data are not publicly available, because the consent obtained at data collection did not cover unrestricted public release; de-identified participant-level files, a variable-level codebook and the analysis code can be shared with qualified researchers, subject to approval by the institutional Research Ethics Committee.

## Supplementary Materials

The following supporting information can be downloaded at: https://www.mdpi.com/article/10.3390/healthcare14172876/s1, Table S1: Internal consistency and sampling adequacy of the study measures in the pilot and main waves; Table S2: Sensitivity analysis excluding respondents with an identical-option CES-D-10 response pattern; Table S3: STROBE Statement—checklist of items that should be included in reports of cross-sectional studies; Table S4: OLS diagnostics and robustness analyses; Figure S1: OLS diagnostic plots for the adjusted CES-D-10 model.

## Abbreviations

The following abbreviations are used in this manuscript:

ANOVA	Analysis of variance
CES-D-10	10-item Center for Epidemiologic Studies Depression Scale
CI	Confidence interval
KAP	Knowledge, attitudes and practices
OR	Odds ratio
SD	Standard deviation
SE	Standard error
STROBE	Strengthening the Reporting of Observational Studies in Epidemiology
VIF	Variance inflation factor
WHO	World Health Organization
