# Supplementary material for: Tobacco-Use Susceptibility and Ever-Use Categories in Relation to Depressive Symptoms Among Undergraduates at a Chinese University: A Cross-Sectional Study
Source: Healthcare (Basel). 2026 Sep 7;14(17):2876. doi: 10.3390/healthcare14172876 (PMC13566508; doi:10.3390/healthcare14172876)
Supplement: Supplementary file 1 [file healthcare-14-02876-s001.zip › healthcare-4528740-supplementary.pdf]

Supplementary Materials

Tobacco-Use Susceptibility and Ever-Use Categories in Relation to Depressive Symptoms Among Undergraduates at a Chinese University: A Cross-Sectional Study

Table S1. Internal consistency and sampling adequacy of the study measures in the pilot and main waves.

Measure (items)	Pilot wave (n = 206) Cronbach's α	Pilot wave KMO	Pilot wave Bartlett's p	Main survey (n = 415) Cronbach's α	Main survey KMO	Main survey Bartlett's p
CES-D-10 depressive symptoms (10)	0.851	0.84	<0.001	0.879	0.88	<0.001
Perceived social support (5)	0.886	0.81	<0.001	0.941	0.89	<0.001
Life satisfaction (single item)	n/a	—	—	n/a	—	—

Note: All indices are computed on the samples stated in the column headings; the main-survey column refers to all 415 respondents, not to the tobacco-naïve subsample. KMO = Kaiser–Meyer–Olkin measure of sampling adequacy; Bartlett = Bartlett's test of sphericity. Cronbach's α for the CES-D-10 is computed on reverse-scored items. KMO and Bartlett's test are not applicable to the single-item life satisfaction measure. Scales collected in the questionnaire but not used in the present analysis are not reported here.

Scope of the pilot wave. The questionnaire was administered in a pilot wave (21 November – 1 December 2025) at the same institution as the main survey (1–2 December 2025), for the purpose of instrument evaluation. The two waves were recruited through the same class-level messaging channels and participation overlapped: at least 43 respondents in each wave could be matched to the other by a distinctive survey-platform display name, and a further subset shared submission IP addresses. Both indicators are released with the data files (overlap_flag_display_name, overlap_flag_ip) so that the extent of overlap can be inspected directly. The waves are therefore reported as partially overlapping rather than independent.

Because internal consistency and factorability describe the behaviour of a set of items rather than the generalisability of a population estimate, these indices remain interpretable under partial overlap. For that reason the pilot wave is used only to document measurement properties. It is not used as an independent replication sample, it was not pooled with the main survey, and no association reported in the main text draws on it; all results in Section 3 are based on the 415 main-survey respondents.

The proportion selecting the same raw response option for all ten CES-D-10 items (straightline_cesd10) was 17.3% in the main survey and 5.8% in the pilot wave. This zero-variation flag is compatible with straightlining or low response differentiation but is not proof of inattention or invalidity. It was not used as a primary exclusion criterion; the post hoc sensitivity analysis is reported in Table S2.

Table S2. Sensitivity analysis excluding respondents with an identical-option CES-D-10 response pattern.

Estimate	Full sample (n = 415)	Pattern-excluded subsample (n = 343)
CES-D-10, mean by tobacco category (0 / 1 / 2)	9.29 / 11.55 / 13.06	8.70 / 10.91 / 12.24
One-way ANOVA across categories	$F(2, 412) = 10.12$, $p < 0.001$, $\eta^2 = 0.047$	$F(2, 340) = 7.27$, $p < 0.001$, $\eta^2 = 0.041$
Tobacco category → CES-D-10, b (95% CI)	1.74 (0.76 to 2.73)	1.73 (0.69 to 2.78)
Tobacco category → CES-D-10, β	0.19	0.19
Social support → CES-D-10, b (95% CI)	−0.36 (−0.52 to −0.20)	−0.63 (−0.82 to −0.44)
Social support → CES-D-10, β	−0.26	−0.41
Model R^2	0.162	0.276
Probable depression (≥10), adjusted OR per one-category increase (95% CI)	1.65 (1.17–2.33)	1.85 (1.22–2.80)
Probable depression (≥12), adjusted OR per one-category increase (95% CI)	1.79 (1.28–2.51)	2.09 (1.37–3.18)
Component a: category → CES-D-10	1.74 (0.76 to 2.73)	1.73 (0.69 to 2.78)
Component b: CES-D-10 → life satisfaction	−0.041 (−0.056 to −0.025)	−0.042 (−0.061 to −0.022)
Total association c	−0.148 (−0.306 to 0.009)	−0.234 (−0.412 to −0.055)
Direct component c′	−0.078 (−0.231 to 0.076), $p = 0.321$	−0.161 (−0.335 to 0.012), $p = 0.067$
Indirect association a × b (bootstrap 95% CI)	−0.071 (−0.124 to −0.027)	−0.072 (−0.132 to −0.022)
Descriptive ratio (a × b)/c; not emphasized	47.7%	30.9%

Note: Regression models were adjusted for sex, academic year, residence, alcohol consumption, physical activity, close friends' smoking, parental smoking, and social support. The one-way ANOVAs were unadjusted. OLS regression-component confidence intervals use HC3 standard errors; the indirect association uses a percentile interval from 5000 nonparametric bootstrap resamples (seed 20260806).

Definition of the exclusion. The flag identifies respondents who selected the same raw response option for all ten CES-D-10 items, producing zero within-person response variation. Because the scale contains both positive- and negative-valence items, the pattern is compatible with straightlining, low response differentiation, misunderstanding, or acquiescence. It is not, by itself, proof of inattention, logical inconsistency, or invalidity. Seventy-two respondents (17.3%) were flagged; their totals take the four arithmetically implied values 6, 12, 18, and 24, and their median completion time was 257 s versus 357 s for other respondents (Mann-Whitney $p < 0.001$). The corresponding proportion in the pilot wave was 5.8%. The released indicator is straightline_cesd10.

Interpretation. The tobacco category–depression association central to this paper was robust: the coefficient was nearly identical (1.74 vs. 1.73), the HC3 intervals excluded zero, the adjusted odds ratios strengthened, and the bootstrap indirect association was stable. The social-support association changed materially (−0.36 vs. −0.63), and the estimated direct/indirect allocation shifted. Under HC3 inference the direct component remained imprecise in both samples ($p = 0.321$ and $p = 0.067$). The manuscript therefore does not claim a stable mediated proportion or a wholly indirect process.

The identical-option pattern was not evenly distributed across tobacco categories (29.8% of ever users, 19.6% of susceptible never users, and 15.1% of firmly non-susceptible never users; χ^2 $p = 0.041$). The pattern-excluded subsample is therefore not a random subset, and these estimates are a robustness check rather than a preferred specification; primary results use all 415 respondents.

Table S3. STROBE Statement—checklist of items that should be included in reports of cross-sectional studies.

Item No.	Recommendation	Reported in
**Title and abstract**		
1	(a) Indicate the study's design with a commonly used term in the title or the abstract	Title ("A Cross-Sectional Study"); Abstract, Methods
	(b) Provide in the abstract an informative and balanced summary of what was done and what was found	Abstract
**Introduction**		
2	Background/rationale: Explain the scientific background and rationale for the investigation being reported	Section 1 (background and rationale).
3	Objectives: State specific objectives, including any prespecified hypotheses	Section 1, final paragraph (primary, secondary, and exploratory aims; H1–H3).
**Methods**		
4	Study design: Present key elements of study design early in the paper	Section 2.1, first sentence
5	Setting: Describe the setting, locations, and relevant dates, including periods of recruitment, exposure, follow-up, and data collection	Section 2.1 (December 2025; public teacher-training university in southeastern China); Section 2.4 (pilot dates).
6	Participants: (a) Give the eligibility criteria, and the sources and methods of selection of participants	Section 2.1 (enrolled undergraduates aged ≥18 years; class groups, university social media, snowball sharing)
7	Variables: Clearly define all outcomes, exposures, predictors, potential confounders, and effect modifiers. Give diagnostic criteria, if applicable	Section 2.2 (three mutually exclusive cross-sectional tobacco categories, CES-D-10, thresholds ≥10/≥12, life satisfaction, support index, and covariates; unmeasured recency and lifetime quantity).
8	Data sources/measurement: For each variable of interest, give sources of data and details of methods of assessment. Describe comparability of assessment methods if there is more than one group	Section 2.2 (self-administered online questionnaire; Pierce susceptibility algorithm; CES-D-10; single-item life satisfaction; five-item support index); Section 2.4 and Table S1 (pilot-wave internal consistency, sampling adequacy and factorability). Identical instrument administered to all participants
9	Bias: Describe any efforts to address potential sources of bias	Sections 2.1 and 2.3 (recruitment, exclusions, covariates, HC3 inference and sensitivity analyses); Section 2.4 (partially overlapping pilot); Sections 3.6 and 4.7 (response-pattern sensitivity and bias limitations).

Item No.	Recommendation	Reported in
10	Study size: Explain how the study size was arrived at	Section 2.1 (492 returned, 77 excluded, 415 retained). No a priori sample-size calculation is reported in the manuscript.
11	Quantitative variables: Explain how quantitative variables were handled in the analyses. If applicable, describe which groupings were chosen and why	Sections 2.2–2.3 (ordinal and indicator category coding; continuous CES-D-10 and ≥10/≥12 thresholds; binary/summed susceptibility; categorical academic year and quadratic support in sensitivity analyses).
12	Statistical methods: (a) Describe all statistical methods, including those used to control for confounding	Section 2.3
	(b) Describe any methods used to examine subgroups and interactions	Sections 2.3, 3.3, and 3.4 (exploratory tobacco-category × social-support interaction; cross-classification of ever use and susceptibility; continuous susceptibility score in the never-user subsample).
	(c) Explain how missing data were addressed	Sections 2.1 and 2.3 (no missing analytic variables in the retained dataset; complete-case primary analyses). The manuscript does not state that the survey used forced responses.
	(d) If applicable, describe analytical methods taking account of sampling strategy	Not applicable — non-probability convenience and chain-referral sample; no weighting applied (Section 2.1; limitation noted in Section 4.7)
	(e) Describe any sensitivity analyses	Sections 2.3 (analyses a–h) and 3.6; Tables S2 and S4; Figure S1.
**Results**		
13	Participants: (a) Report numbers of individuals at each stage of the study	Section 2.1 (492 returned → 77 excluded → 415 analyzed)
	(b) Give reasons for non-participation at each stage	Section 2.1 (41 completion time <3 min; 25 non-undergraduate; 11 with >50% missing tobacco items). A denominator of invited students could not be defined under open chain-referral recruitment
	(c) Consider use of a flow diagram	Participant flow is reported in text (Section 2.1); a diagram was not considered necessary given the single-stage design
14	Descriptive data: (a) Give characteristics of study participants and information on exposures and potential confounders	Table 1; Section 3.1
	(b) Indicate number of participants with missing data for each variable of interest	Sections 2.1 and 2.3 (none in retained analytic variables; N = 415 in primary models, with subgroup and sensitivity denominators specified separately).

Item No.	Recommendation	Reported in
15	Outcome data: Report numbers of outcome events or summary measures	Sections 3.1–3.2; Table 2 (overall CES-D-10 10.03 ± 6.14; probable depression 211/415 [50.8%]; group CES-D-10, depression prevalence, and life satisfaction). Overall social support is reported in Section 3.1.
16	Main results: (a) Give unadjusted estimates and, if applicable, confounder-adjusted estimates and their precision. Make clear which confounders were adjusted for and why they were included	Tables 2–5; Sections 3.2–3.5. Unadjusted group means and trend tests in Table 2; adjusted coefficients with 95% CIs in Tables 3–5; covariate selection rationale in Section 2.2
	(b) Report category boundaries when continuous variables were categorized	Section 2.2 (Levels 0/1/2 defined by susceptibility and ever-use responses; CES-D-10 thresholds ≥10 and ≥12).
	(c) If relevant, consider translating estimates of relative risk into absolute risk for a meaningful time period	Absolute prevalence of probable depression by tobacco category is reported alongside the adjusted odds ratio (Table 2, Figure 1B, Section 3.3)
17	Other analyses: Report other analyses done—e.g., analyses of subgroups and interactions, and sensitivity analyses	Sections 3.3–3.6 (interaction, four-group and never-user analyses, product-specific definitions, additional adjustment, ≥12 threshold, response-pattern and duration exclusions, and information-channel model); Tables S2/S4; Figure S1.
**Discussion**		
18	Key results: Summarise key results with reference to study objectives	Section 4, opening paragraph
19	Limitations: Discuss limitations of the study, taking into account sources of potential bias or imprecision. Discuss both direction and magnitude of any potential bias	Section 4.7 (cross-sectional design, non-probability recruitment, threshold, measurement, response-process uncertainty, model assumptions, subgroup imprecision, and unmeasured confounding).
20	Interpretation: Give a cautious overall interpretation of results considering objectives, limitations, multiplicity of analyses, results from similar studies, and other relevant evidence	Sections 4.1–4.6; explicit non-causal framing in Sections 2.3, 3.5, 4.1 and 4.3
21	Generalisability: Discuss the generalisability (external validity) of the study results	Section 4.7 (single teacher-training university, 75.4% female, convenience and snowball recruitment)
**Other information**		
22	Funding: Give the source of funding and the role of the funders for the present study and, if applicable, for the original study on which the present article is based	Funding statement (funders, grant numbers and APC); Conflicts of Interest (funder role); Section 2.1 (prior reports [20,21]).

Source: the STROBE statement, Reference [29] in the main manuscript. The checklist retains the supplied recommendations and updates the reporting locations; it does not imply that unreported procedures were performed. https://www.strobe-statement.org/

Table S4. OLS diagnostics and robustness analyses.

The primary coefficient is the adjusted mean CES-D-10 difference per one-category increase in the cross-sectional tobacco uptake/susceptibility variable. HC3 = heteroscedasticity-consistent covariance estimator; RESET = regression-equation specification error test; VIF = variance inflation factor.

Diagnostic / sensitivity	Result	Interpretation
Breusch-Pagan test	F = 1.85; p = 0.058	Borderline evidence of heteroscedasticity
White test	F = 1.70; p = 0.004	Evidence of heteroscedasticity; HC3 inference used
Ramsey RESET, linear Model 3	F = 11.05; p = 0.001	Functional-form departure detected
Residual distribution	Jarque-Bera = 17.46; p < 0.001; skew = 0.50	Mild right-skew; one external studentized residual > |3|
Collinearity	Maximum VIF = 1.23	No material collinearity
Leverage	5 > 2p/N = 0.048; maximum = 0.062	A small number of high-leverage observations
Cook's distance	26 > 4/N = 0.0096; maximum = 0.037	No observation approached Cook's D = 1
Primary OLS coefficient with HC3	b = 1.74 (95% CI 0.76 to 2.73)	Core category association retained
Flexible functional form	b = 1.70 (HC3 95% CI 0.74 to 2.67); RESET p = 0.840	Categorical academic year plus quadratic support removed RESET signal
Exclude Cook's D > 4/N	n = 389; b = 1.90 (95% CI 1.11 to 2.69)	Influence sensitivity retained association
Exclude most influential observation	n = 414; b = 1.79 (95% CI 0.91 to 2.68)	No single observation drove the estimate
Exclude fastest 5%	n = 393; b = 1.71 (HC3 95% CI 0.69 to 2.73)	Duration sensitivity retained association
Exclude fastest 10%	n = 371; b = 2.04 (HC3 95% CI 1.02 to 3.06)	Duration sensitivity retained association
Exclude identical-option pattern	n = 343; b = 1.73 (HC3 95% CI 0.69 to 2.78)	Response-pattern sensitivity retained association

Figure S1. OLS diagnostic plots for the adjusted CES-D-10 model.

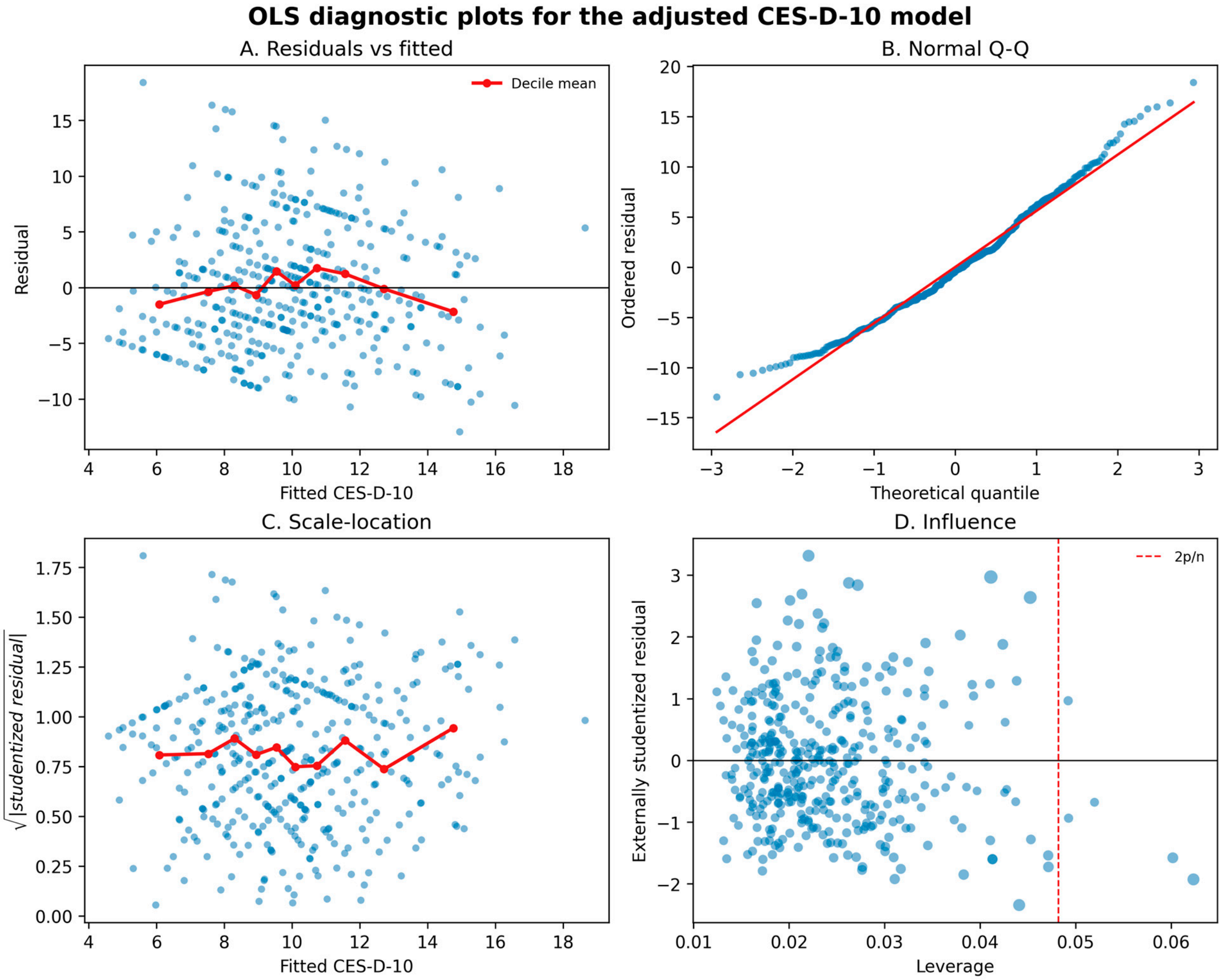

Panel A shows residuals versus fitted values with decile means; Panel B shows the normal Q-Q plot; Panel C shows the scale-location plot; Panel D shows externally studentized residuals against leverage, with point size proportional to Cook's distance. The plots and formal tests motivated HC3 inference and the flexible functional-form sensitivity analysis in Table S4.
